# State Registration of Trained Nurses

**Published:** 1919-03-29

**Authors:** 


### STATE REGISTRATION OF TRAINED NURSES.

**A Statement in support of the Bill promoted by the College of Nursing, Ltd., as against the Bill of the Central Committee, to be introduced in the House of Commons on Friday, March 28, 1919.**

**[The following statement has been sent to every Member of Parliament.]**

THE COLLEGE OF NURSING.

1. *The College of Nursing, Ltd., already possesses a Register of over 12,600 trained nurses,* and the Bill promoted by it and repeatedly revised after careful consideration of the Central Committee's Bill is believed to offer a scheme of registration for nurses more in harmony with the recent developments which have marked the progress of women, and free from the rigidity which characterises the older Bill which would prevent adaptation to altered conditions without an amending Act. A few essentials excepted, all regulations made under the College Bill may be changed as circumstances require by the authority of the Privy Council, after being laid before both Houses of Parliament.

FUNDS.

2. *The College possesses funds* to the value of over £40,000, which the College Bill places at the disposal of the General Nursing Council to be used for the benefit of all registered nurses.

CHARACTER.

3. *The College of Nursing* is a Company limited by guarantee not having a share capital, and claims in the Bill to be allowed to drop the word "limited," being in fact already precluded from making a profit or paying dividends.

PROGRESS.

4. *The College of Nursing* has achieved in three years a result which the promoters of the Central Committee's Bill for the State Registration of Nurses have failed to achieve during the long period of their activity both before and since the formation of that body in 1909. Even now it is impossible to obtain any trustworthy information as to the actual number of trained nurses who belong to the much-quoted "Organised Nurses' Societies." Many nurses certainly belong to three of them, and totals are thus inflated.

### The Bills Contrasted.

5. *The College Bill is shorter.* It deals with essentials only, and leaves details to be settled by the Privy Council.

6. *The scope of the Central Committee's Bill* is limited to securing for the nurse the right to call herself a registered nurse and to have her name on a State Register.

PROGRAMME COLLEGE OF NURSING.

*The College also aims at State Registration,* with uniformity of standard and the one-portal system, but only as an instalment of its wider programme for the betterment of nursing conditions, social, professional, and economic, included in its Memorandum of Association.

REPRESENTATION ON GENERAL NURSING COUNCIL.

7. (*a*) *In the Central Committee's Bill* it is proposed to set up a Council of forty-one members, of whom eighteen are to be nurses on the General Register, and of these trained nurses practically half must be past or present matrons. Is this limitation fair to the rank-and-file of the profession? **In the College Bill not less than two-thirds of the Council** are elected by the trained nurses, and of the persons so elected *five-sixths* must themselves be trained nurses.

(*b*) **In the Central Committee's Bill** the representation of the various parts of the United Kingdom is gravely disproportionate, *e.g.*, **England has eight and Ireland four Nurses on the Council. The College Bill gives four-sixths to England and Wales, one-sixth to Scotland, and one-sixth to Ireland.**

(*c*) *In the Central Committee's Bill* no representation on the Provisional Nursing Council is accorded to the managers of voluntary hospitals or Poor-Law infirmaries, and on the General Nursing Council Ireland has no less than half the number of representatives of the nurses' training-schools assigned to England and Wales.

PROVISIONAL NURSING COUNCIL.

8. On the first *Provisional Nursing Council* the College Bill concedes to the Central Committee the same number of representatives as it claims for itself. **The Central Committee's Bill gives the College and its 12,600 members two places out of twenty-eight.**

9. *The Central Committee's Bill* keeps the Provisional Nursing Council in office for two years or more, and allows this provisional, non-elected, Council to make the rules under which the nursing profession has to live. *The College Bill* allows the Provisional Council merely to prepare a scheme for setting up the General Nursing Council, which scheme has then to be approved by the Privy Council That done, the Provisional Council makes way for the General Nursing Council.

SUPPLEMENTARY REGISTERS.

10. *The Central Committee's Bill* admits the necessity for Supplementary Registers, but limits them to male and mental nurses. The *College Bill* enacts that there may also be supplementary registers in other branches of the profession—*e.g.* children's nurses.

**Finance.**

11. **The Central Committee's Bill fails entirely to provide any financial basis** adequate to sustain the expenses involved in:—

(*a*) Conducting examinations during their three years' training for all the nurses at the various recognised hospitals and infirmaries in the United Kingdom. (*b*) Publishing (annually) the Register, which must include the names and addresses of many thousands of trained nurses. In this connection the College is expending more than £1,000 in publishing its own first Register of 12,600 nurses. (*c*) Erecting or renting buildings suitable for central offices, an examination bureau, a registration and filing department, etc. (*d*) Paying examiners, inspectors of nurse-training schools, secretaries, registrars, and clerical staff. (*e*) Providing premises, equipment, and staffs for divisional boards. (*f*) Paying travelling expenses, and fees for attendance, for members of Council (forty-one). Towards these expenses—and the list might be amplified—*the only available income is the fees paid by nurses* for examination and registration, and no *educational institution can pay its way on students' fees alone.*

[A copy of the Bill drafted by the College of Nursing, Limited, may be obtained from Messrs. Eyre & Spottiswoode, Ltd., East Harding Street, E.C. Price Threepence.]

